# Comparison of the efficacy and safety of different growth factors in the treatment of diabetic foot ulcers: an updated network meta-analysis

**DOI:** 10.3389/fendo.2025.1614597

**Published:** 2025-06-25

**Authors:** Jianzhou Tian, Guanghui Yao, Tian Tian, Xinlin Li, Shaoru Li, Chengda Wu, Saisheng Zhang

**Affiliations:** ^1^ Department of Burn and Plastic Surgery, Renmin Hospital, Hubei University of Medicine, Shiyan, Hubei, China; ^2^ Reproductive Medicine Center, Renmin Hospital, Hubei University of Medicine, Shiyan, Hubei, China; ^3^ Hubei Clinical Research Center for Reproductive Medicine, Shiyan, Hubei, China; ^4^ Shiyan Key Laboratory of Reproduction and Genetics (Renmin Hospital, Hubei University of Medicine), Shiyan, Hubei, China; ^5^ Biomedical Engineering College, Hubei University of Medicine, Shiyan, China; ^6^ Department of Ophthalmology, Renmin Hospital, Hubei University of Medicine, Shiyan, Hubei, China

**Keywords:** diabetic foot ulcer, growth factors, standard of care, randomized controlled trial, network meta-analysis

## Abstract

**Objective:**

This study aimed to evaluate the efficacy and safety of different growth factors (GFs) in the treatment of diabetic foot ulcers (DFUs) through a network meta-analysis.

**Methods:**

A systematic search was conducted in PubMed, Embase, The Cochrane Library, and Web of Science to identify randomized controlled trials (RCTs) comparing GFs with standard of care (SOC) or comparing different GFs for the treatment of DFU. Two independent reviewers screened the studies, extracted data, and assessed the quality of the included literature according to predefined inclusion and exclusion criteria. A network meta-analysis was performed using R software. Relative risk (RR) was used as the effect measure for dichotomous outcomes, and mean difference (MD) was used for continuous outcomes.

**Results:**

A total of 51 RCTs, involving 3,401 patients with DFUs and six different types of GFs, were included. The network meta-analysis revealed that, compared with SOC, epidermal growth factor (EGF), platelet-derived growth factor (PDGF), and platelet-rich plasma (PRP) significantly improved the healing rate. EGF and PRP also significantly reduced healing time, while PDGF significantly reduced ulcer area. Moreover, PRP was associated with a significant reduction in the incidence of adverse events (AEs) and amputation rates. In terms of ranking: For healing rate, the top three GFs were EGF, vascular endothelial growth factor (VEGF), and granulocyte colony-stimulating factor (G-CSF). For healing time, EGF, PRP, and fibroblast growth factor (FGF) ranked the highest. For ulcer area reduction, PDGF, EGF, and PRP were the top-ranking interventions. Regarding AEs, PRP, PDGF, and FGF showed the most favorable safety profiles. For amputation rate, PRP, G-CSF, and PDGF were ranked the highest.

**Conclusion:**

Almost all GFs outperformed SOC in terms of healing rate, healing time, and ulcer area reduction. Compared to SOC, EGF, PDGF, and PRP significantly improved healing rates; EGF and PRP significantly reduced healing time; and PDGF significantly decreased ulcer area. Among them, EGF may be the most effective GF. Except for VEGF, which significantly increased AEs, other GFs did not show a significant increase in AEs compared to SOC. PRP had the lowest amputation rate and incidence of AEs.

**Systematic review registration:**

https://www.crd.york.ac.uk/prospero/, identifier CRD420251035765

## Introduction

Diabetic foot ulcer (DFU) is a common and serious complication in patients with diabetes, characterized by a high recurrence rate and associated with increased amputation and mortality rates (1). Currently, approximately 18.6 million people worldwide are affected by DFU (2). With the continuous rise in diabetes prevalence, the incidence of DFU has also shown a significant increase, placing a heavy economic burden on patients, their families, and society (3). Wound healing is frequently compromised in patients with DFUs, and their clinical condition can easily worsen (4). Common standard of care (SOC) treatments such as debridement, dressing changes, pressure relief, and blood glucose control have limited efficacy (5). Therefore, there is an urgent need for new treatment options.

Growth factors (GFs) play a crucial role in the wound healing process. Platelet-derived growth factor (PDGF), platelet-rich plasma (PRP), epidermal growth factor (EGF), fibroblast growth factor (FGF), vascular endothelial growth factor (VEGF), and granulocyte colony-stimulating factor (G-CSF) are among the most extensively studied GFs in the treatment of DFU. PDGF facilitates cell recruitment and tissue repair and has been approved by the Food and Drug Administration (FDA); PRP, rich in multiple GFs, accelerates wound healing and is increasingly used in clinical practice; EGF promotes keratinocyte proliferation and migration, enhancing wound healing; FGF supports granulation tissue formation and collagen synthesis, though its application remains limited; VEGF improves local perfusion through angiogenesis but is less commonly used clinically; and G-CSF enhances immune function, although related research is relatively limited (6). Several direct meta-analyses have shown that GFs can significantly improve DFU healing compared to standard treatment (7–9). However, the International Working Group on the Diabetic Foot believes that the existing evidence is insufficient to support the use of GFs in the treatment of DFU (10). Since current studies mainly focus on comparing the efficacy of a single GF with SOC, there is a lack of evidence on the differences in efficacy and safety between different GFs for treating DFU. Two previous network meta-analyses have both suggested that EGF is the most effective GF for healing DFU (11, 12). However, the outcome measures reported in these studies were limited to healing rate, without addressing other important outcomes such as healing time, ulcer area reduction, and the incidence of adverse events (AEs). Additionally, clinical research on GF treatment for DFU is continuously being updated (6). Therefore, an updated network meta-analysis is needed to evaluate the efficacy and safety of different GFs in the treatment of DFU, with the aim of providing more evidence-based medical support for DFU treatment.

## Methods

This study is reported in accordance with the Preferred Reporting Items for Systematic Reviews and Meta-Analyses (PRISMA) guidelines (13). The study has been registered in PROSPERO with the registration number CRD420251035765.

### Literature search strategy

PubMed, Embase, The Cochrane Library, and Web of Science were searched to identify randomized controlled trials (RCTs) comparing GFs with SOC, or comparing different GFs for the treatment of DFU. The search was conducted from the inception of each database to 12 January 2025. A combination of subject terms and free-text terms was used for the search. Detailed search strategies for each database are provided in **Supplementary file S1**.

### Inclusion criteria

(1) The study participants were patients with DFU. (2) The study design was an RCT. (3) The study included two or more comparison groups. (4) The interventions included one group receiving SOC and the other group(s) receiving GF treatment, or different groups receiving different types of GFs. (5) The study reported at least one of the following outcome measures: healing rate, healing time, ulcer area reduction, amputation rate, or AEs. AEs included events such as infection, allergic reactions, and pain.

### Exclusion criteria

The following types of studies were excluded: (1) studies from which valid data could not be extracted and for which attempts to contact the authors were unsuccessful; (2) conference abstracts and letters; (3) publications derived from the same study population; (4) single-arm studies, case reports, retrospective studies, and other non-randomized designs; and (5) studies published in languages other than English.

### Study selection, data extraction, and quality assessment

(1) Two researchers independently screened the literature, extracted relevant data, and assessed the methodological quality of the included studies based on pre-defined inclusion and exclusion criteria. The results were cross-checked between the two reviewers. Any discrepancies that could not be resolved through discussion were adjudicated by a third reviewer. (2) Extracted data included the following: basic study information such as first author, year of publication, and study location; patient-related information such as interventions, the number of male and female patients in each group, age, duration of ulcers, and ulcer area; and outcome-related data including healing rate, healing time, ulcer area reduction, amputation rate, and incidence of AEs. If a study involved different doses of the same GF, data from the group receiving the highest dose were used. Relevant elements for risk of bias assessment were also extracted. (3) The risk of bias of the included RCTs was assessed using the Risk of Bias 2.0 (RoB 2.0) tool (14).

### Statistical analysis

A network meta-analysis was performed using the Gemtc package in R (15). Network evidence diagrams were generated for each outcome measure. A Bayesian statistical approach was employed to conduct indirect comparisons of the efficacy and safety of different GFs in the treatment of DFU. For healing rate and incidence of AEs, relative risk (RR) was used as the effect measure; for healing time and ulcer area reduction, mean difference (MD) was used. When the interventions related to the outcome indicators do not form a closed loop, the assumption of consistency is satisfied. If the interventions form a closed loop, a node-splitting method is used to assess consistency; a *p*-value greater than 0.05 suggests an acceptable level of consistency between direct and indirect evidence. A Bayesian Markov Chain Monte Carlo (MCMC) random-effects model was used for the analysis (16). Model parameters were set as follows: four chains, 50,000 iterations, and 20,000 burn-ins. Effect sizes and their 95% credible intervals (CIs) were calculated for each outcome across the interventions. The efficacy and safety of different treatments were compared, and the surface under the cumulative ranking curve (SUCRA) was used to rank the treatments in terms of efficacy and safety (17). Sensitivity analysis was performed by excluding studies with a high risk of bias.

## Results

### Results of literature search

**Figure 1:**
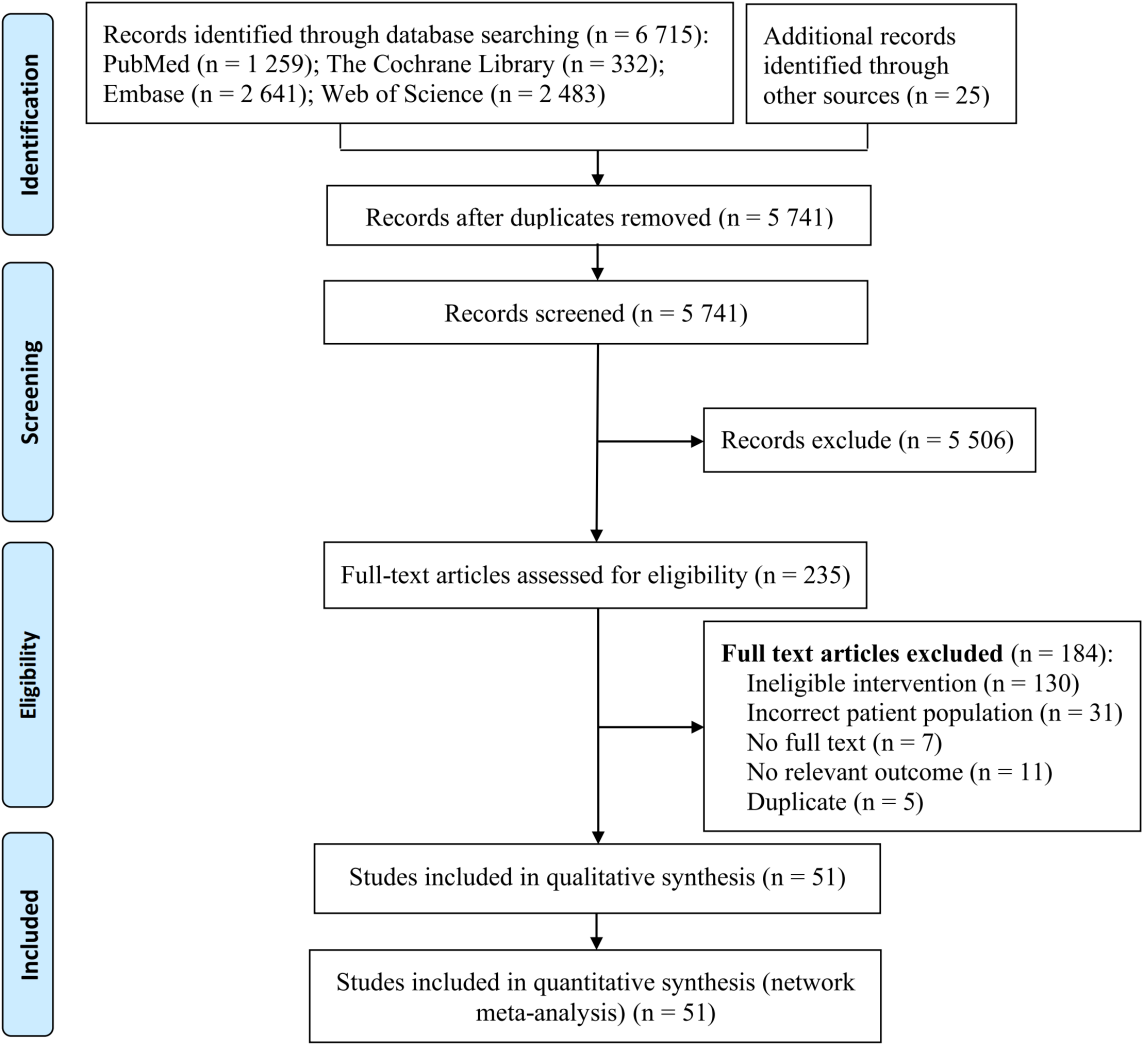
Literature screening process and results.

A total of 6,740 relevant studies were identified through a systematic search of the databases. After rigorous screening, 51 studies (18–68) were found to meet the inclusion criteria. The detailed process and results of study selection are illustrated in **Figure 1**.

### Basic characteristics of included studies

A total of 51 RCTs, involving 3,401 patients with DFU, were included. These studies examined seven different interventions, namely, EGF, VEGF, G-CSF, FGF, PDGF, PRP, and SOC. Detailed characteristics of the included studies are presented in **Table 1**.

**Table 1:** The characteristics of included studies on the treatment of diabetic foot ulcers.

Author and year	Country	Group	Number of patients	Mean age (years)	Male gender (*n*)	Wound duration (weeks)	Wound area (cm^2^)	Study period (weeks)	Outcomes
Steed DL 1992 (18)	USA	PRP	7	58.7 ± 12.4	5/2	68	NG	20	Healing rate, ulcer reduction area
		SOC	6	54.2 ± 12.9	4/2	52	NG		
Richard JL 1995 (19)	France	FGF	9	61.9 ± 10.0	9/0	89.6 ± 111.6	NG	12	Healing rate, ulcer reduction area, AEs
		SOC	8	63.6 ± 7.9	7/1	111.6 ± 168.8	NG		
Wieman TJ 1998 (20)	USA	PDGF	61	63.2	43/18	81.8 (6.6–536.0)	5.5	20	Healing rate, AEs
		SOC	57	58.3	46/11	74.5 (6.7–349.6)	9		
Smiell JM 1998 (21)	USA	PDGF	123	57.0 ± 11.5	82/41	46.0 ± 54.7	2.6 ± 3.4	20	Healing rate, AEs
		SOC	127	58.0 ± 11.8	91/36	46.0 ± 52.1	2.8 ± 4.1		
Yönem A 2000 (22)	Turkey	G-CSF	15	60.4 ± 1.3	8/7	NG	NG	2	Amputation rate
		SOC	15	61.1 ± 1.4	9/6	NG	NG		
de Lalla F 2001 (23)	Italy	G-CSF	20	8.6 (42.0–74.0)	16/4	NG	NG	24	Healing rate, amputation rate
		SOC	20	9.6 (44.0–85.0)	14/6	NG	NG		
Tsang MW 2003 (24)	China	EGF	21	62.2 ± 13.7	6/15	11.5 ± 14.7	3.4 ± 1.1	24	Healing rate, amputation rate
		SOC	19	64.4 ± 11.7	10/9	12.0 ± 15.5	3.5 ± 0.8		
Kästenbauer T 2003 (25)	Austria	G-CSF	20	60.8 ± 11.1	15/5	NG	NG	2	Amputation rate
		SOC	17	58.2 ± 8.1	13/4	NG	NG		
Saldalamacchia G 2004 (26)	Italy	PRP	7	61.1 ± 9.4	4/3	NG	2.7 ± 1.6	5	Ulcer reduction area
		SOC	7	58.1 ± 7.8	2/5	NG	1.7 ± 0.9		
Huang P 2005 (27)	China	G-CSF	14	71.1 ± 5.9	9/5	NG	2.7 ± 1.3	12	Healing rate, amputation rate
		SOC	14	70.9 ± 6.0	9/5	NG	2.4 ± 1.2		
Robson M 2005 (28)	USA	PDGF	74	NG	NG	NG	NG	20	Healing rate
		SOC	72	NG	NG	NG	NG		
Afshari M 2005 (29)	Iran	EGF	30	56.9 ± 12.7	16/14	0.9 ± 1.4	0.9 ± 1.1	4	Healing rate
		SOC	20	59.7 ± 12.3	11/9	2.1 ± 2.0	1.0 ± 1.4		
Driver VR 2006 (30)	USA	PRP	19	58.3 ± 9.7	16/3	4.0	3.4 ± 4.5	12	Healing rate, healing time, ulcer reduction area
		SOC	21	55.9 ± 8.1	16/5	4.0	3.6 ± 4.0		
Hanft JR 2008 (31)	USA	VEGF	29	59.5 (42.0–74.0)	19/10	NG	1.9 (1.0–4.1)	6	Healing rate, AEs
		SOC	26	59.3 (38.0–81.0)	18/8	NG	1.9 (1.1–2.9)		
Fernández JI 2009 (32)	Cuba	EGF	53	63.0 (55.0–69.0)	28/25	4.3 (2.9–10.3)	28.5(10.4–42.8)	8	Healing rate, healing time, AEs, amputation rate
		SOC	48	64.0 (51.0–70.0)	27/21	4.9 (3.3–12.9)	21.8 (8.8–34.6)		
Bhansali A 2008 (33)	India	PDGF	10	51.7 ± 13.6	7/3	NG	18.1 ± 15.9	20	Healing rate, healing time
		SOC	10	49.5 ± 8.8	5/5	NG	11.1 ± 9.3		
Agrawal R 2009 (34)	Iran	PDGF	14	54.4 ± 8.8	9/5	NG	55.6 ± 4.5	12	Healing rate, ulcer reduction area
		SOC	14	56.2 ± 8.8	10/4	NG	33.8 ± 2.5		
Landsman A 2010 (35)	Israel	PDGF	16	58.1	NG	NG	3.8	20	Healing rate
		SOC	16	56.2	NG	NG	5.6		
Jaiswal SS 2010 (36)	India	PDGF	25	56.2 ± 11.3	19/6	5	30.0 ± 3.5	10	Healing rate
		SOC	25	49.9 ± 18.9	23/2	6	26.5 ± 2.5		
Khandelwal S 2013 (37)	India	PDGF	20	43.4 ± 8.1	11/9	NG	19.3 ± 11.3	10	Healing rate, healing time, ulcer reduction area
		SOC	20	45 ± 7.6	11/9	NG	9.9 ± 5.6		
Singla S 2014 (38)	India	EGF	25	58.8	21/4	NG	19.6	8	Healing rate, amputation rate
		SOC	25	22.8	23/2	NG	21.2		
Gomez-Villa R 2014 (39)	Mexico	EGF	17	62.1 ± 12.8	9/8	25.8 ± 44.0	19.2 ± 15.7	8	Healing rate, ulcer reduction area
		SOC	17	19.2 ± 15.7	12/5	36.5 ± 75.8	11.9 ± 11.8		
Ma C 2015 (40)	USA	PDGF	23	59.3 ± 6.7	23/0	18.5 ± 22.2	2.6 ± 2.7	16	Healing rate, amputation rate
		SOC	23	60.1 ± 9.2	23/0	9.8 ± 11.6	3.1 ± 3.4		
Li L 2015 (41)	China	PRP	59	61.4 ± 13.1	37/22	4.3 ± 2.7	4.1 ± 2.5	12	Healing rate, healing time, amputation rate
		SOC	58	34.1 ± 9.4	38/20	3.3 ± 1.6	2.9 ± 2.4		
Liu G 2016 (42)	China	PRP	30	54.6 ± 9.6	18/12	1.0 ± 0.4	4.7 ± 1. 4	8	Healing rate, healing time
		FGF	30	55.4 ± 8.2	16/14	1.1 ± 0.5	5.1 ± 1.9		
Karimi R 2016 (43)	Iran	PRP	25	NG	20/5	NG	NG	4	Healing rate, ulcer reduction area
		SOC	25	NG	18/7	NG	NG		
Antony 2016 (44)	India	EGF	30	20.0–70.0	NG	NG	NG	18	Healing rate
		SOC	30	20.0–70.0	NG	NG	NG		
Samuel A 2016 (45)	India	PDGF	29	56.1	17/12	15.4 ± 15.5	31.4 ± 61.4	24	Healing rate
		SOC	29	56.1	17/12	15.4 ± 15.5	31.4 ± 61.4		
Ahmed M 2016 (46)	Egypt	PRP	28	43.2 ± 18.2	20/8	12.5 ± 1.0	2.5–11.6	12	Healing rate, ulcer reduction area, AEs
		SOC	28	49.8 ± 15.4	18/10	11.5 ± 2.8	2.2–10.2		
Yang L 2017 (47)	China	PRP	38	40.1 ± 10.2	17/21	NG	NG	4	Healing time
		SOC	38	43.7 ± 9.8	19/19	NG	NG		
Singh SP 2018 (48)	India	PRP	29	53.8 ± 10.4	19/10	NG	NG	4	Healing rate, healing time, AEs, amputation rate
		SOC	26	55.6 ± 10.4	15/11	NG	NG		
Xu J 2018 (49)	China	EGF	50	65.0 ± 3.7	25/25	16.0 ± 0.6	4.7 ± 0.3	8	Healing time
		FGF	50	60.0 ± 6.2	24/26	14.0 ± 0.3	5.1 ± 0.2		
		SOC	49	63.0 ± 4.6	25/24	13.0 ± 0.4	4.2 ± 0.4		
Park KH 2018 (50)	Korea	EGF	82	56.6 ± 12.7	55/27	38.5 ± 70.6	2.8 ± 3.7	12	Healing rate, ulcer reduction area, AEs, amputation rate
		SOC	85	59.3 ± 12.6	49/36	29.6 ± 60.2	2.4 ± 2.7		
David TD 2018 (51)	India	EGF	25	25.0–75.0	20/5	NG	NG	4	Healing rate, ulcer reduction area
		SOC	25	25.0–75.0	19/6	NG	NG		
Rainys D 2019 (52)	Lithuania	PRP	35	62.2 ± 14.7	18/17	NG	12.9 ± 16.6	8	Healing rate, ulcer reduction area, AEs
		SOC	34	68.0 ± 14.9	17/17	NG	10.4 ± 11.3		
Gude W 2019 (53)	USA	PRP	66	64.7	51/15	NG	4.1	12	Healing rate, amputation rate
		SOC	63	66.9	49/14	NG	5.6		
Viswanathan V 2019 (54)	India	EGF	27	54.9 ± 2.4	15/12	NG	9.1 ± 9.5	4	Healing rate, healing time
		SOC	23	54.8 ± 3.9	12/11	NG	8.4 ± 7.9		
Elsaid A 2019 (55)	Egypt	PRP	12	54.7 ± 6.6	8/4	21.0 ± 13.6	NG	20	Healing time, ulcer reduction area
		SOC	12	55.6 ± 6.5	6/6	22.3 ± 10.8	NG		
Xie J 2019 (56)	China	PRP	25	60.5 ± 8.3	14/11	3.1 ± 2.6	11.8 ± 9.7	8	Healing rate, ulcer reduction area
		SOC	23	61.1 ± 7.9	13/10	3.5 ± 2.4	11.8 ± 7.8		
Oliveira BC 2021 (57)	Brazil	EGF	14	60.6 ± 8.6	NG	NG	NG	12	Healing rate, ulcer reduction area
		SOC	11	65.1 ± 6.5	NG	NG	NG		
Malekpour AN 2021 (58)	Iran	PRP	43	56.3 ± 7.1	26/17	NG	NG	24	Healing time, amputation rate
		SOC	47	56.7 ± 7.2	30/17	NG	NG		
Habeeb T 2021 (59)	Egypt	PRP	22	57.0 ± 8.1	16/6	NG	NG	12	Healing rate, healing time, ulcer reduction area
		SOC	22	40.0 ± 7.2	16/6	NG	NG		
Gupta A 2021 (60)	India	PRP	30	56.0 ± 9.6	22/8	13.7 ± 17.6	5.2 ± 3.8	6	Healing rate, ulcer reduction area
		SOC	30	55.8 ± 10.2	19/11	11.2 ± 17.7	5.0 ± 2.9		
Hossam EM 2022 (61)	Egypt	PRP	40	54.9 ± 2.4	28/12	12	15.2 ± 5.6	8	Ulcer reduction area, AEs, amputation rate
		SOC	40	54.8 ± 3.9	34/6	12	14.5 ± 5.6		
Mandadap S 2022 (62)	India	PRP	24	41.0–50.0	15/9	NG	NG	10	Healing rate
		SOC	24	51.0–60.0	18/6	NG	NG		
Mohammadi TA 2022 (63)	Iran	PDGF	81	55.8 ± 5.6	52/29	6.0 ± 0.7	3.2 ± 0.5	10	Ulcer reduction area, amputation rate
		SOC	80	60.2 ± 5.2	46/35	6.4 ± 1.8	3.3 ± 0.5		
Zhao P 2023 (64)	China	PRP	15	51.9 ± 8.4	9/6	NG	10.1 ± 2.7	3	Healing rate, healing time, ulcer reduction area
		SOC	15	54.1 ± 7.4	7/8	NG	12.1 ± 3.7		
Satapathy A 2023 (65)	India	PRP	36	NG	NG	NG	10.1 ± 8.8	4	Ulcer reduction area
		SOC	36	NG	NG	NG	9.5 ± 8.7		
Kamineni R 2023 (66)	India	PRP	32	NG	22/10	NG	NG	4	Healing rate, healing time, ulcer reduction area
		SOC	32	NG	24/8	NG	NG		
Abhirami C 2023 (67)	India	PRP	21	51.0–61.0	16/5	NG	11.0 ± 4.4	5	Healing rate, ulcer reduction area
		SOC	21	51.0–61.0	14/7	NG	10.6 ± 4.8		
Gowsick S 2023 (68)	India	PRP	87	NG	50/37	NG	0.5 ± 0.1	12	Healing rate
		SOC	87	NG	54/33	NG	0.5 ± 0.1		

PRP, platelet-rich plasma; SOC, standard of care; FGF, fibroblast growth factor; PDGF, platelet-derived growth factor; G-CSF, granulocyte colony-stimulating factor; EGF, epidermal growth factor; VEGF, vascular endothelial growth factor; AEs, adverse events.

### Quality assessment

The risk of bias assessment showed that 1 study was judged to have a low risk of bias, 46 studies were judged to have some concerns, and 4 studies were judged to have a high risk of bias. Overall, the methodological quality of the included studies was relatively low. Detailed results of the risk of bias assessment are presented in **Supplementary Figure S1**.

### Network evidence diagrams

**Figure 2:**
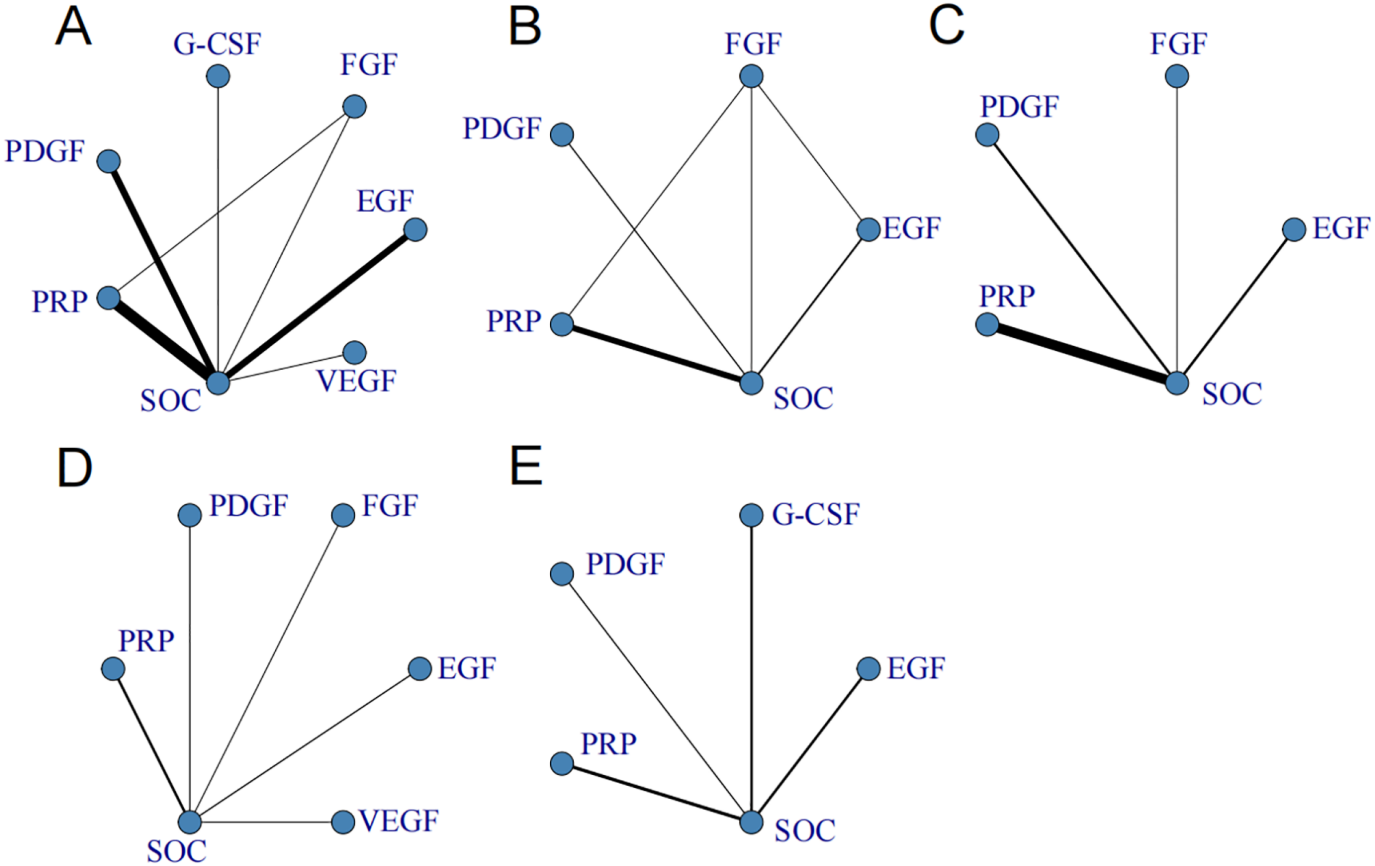
Network evidence plots for **(A)** healing rate, **(B)** healing time, **(C)** ulcer area reduction, **(D)** incidence of adverse events, and **(E)** amputation rate. PRP, platelet-rich plasma; SOC, standard of care; FGF, fibroblast growth factor; PDGF, platelet-derived growth factor; G-CSF, granulocyte colony-stimulating factor; EGF, epidermal growth factor; VEGF, vascular endothelial growth factor.

**Figures 2A–E** present the network evidence diagrams for healing rate, healing time, ulcer area reduction, incidence of AEs, and amputation rate, respectively. In these diagrams, each node represents an intervention. The lines connecting the nodes indicate the presence of direct comparison evidence, and the thickness of the lines is proportional to the number of studies included in the comparison. In the network plots of healing rate and healing time, closed loops were formed among the interventions (consistency assessed using the node-splitting method, *p* > 0.05), while no closed loops were observed for the remaining outcome indicators, suggesting acceptable consistency across the studies.

### Healing rate

**Figure 3:**
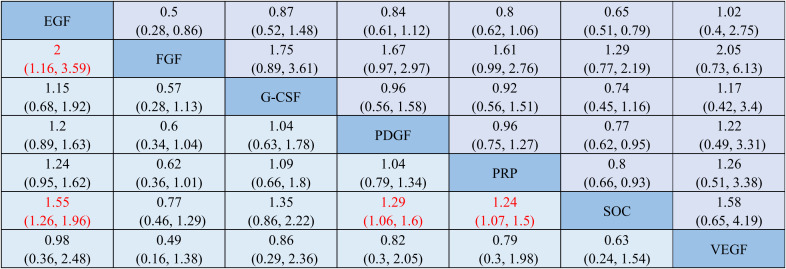
League table of pairwise comparisons for healing rates among different treatment interventions. Each cell presents the relative risk (RR) and 95% confidence interval (CI) for the treatment listed in the column compared with the treatment listed in the row. If the RR is greater than 1 and the difference is statistically significant, the treatment in the column is superior to the treatment in the row. Statistically significant results (*p* < 0.05) are highlighted in bold red font. PRP, platelet-rich plasma; SOC, standard of care; FGF, fibroblast growth factor; PDGF, platelet-derived growth factor; G-CSF, granulocyte colony-stimulating factor; EGF, epidermal growth factor; VEGF, vascular endothelial growth factor.

Compared to SOC, EGF (RR = 1.55, 95% CI = 1.26–1.96), PDGF (RR = 1.29, 95% CI = 1.06–1.60), and PRP (RR = 1.24, 95% CI = 1.07–1.50) significantly improved the healing rate, with statistically significant differences. Among comparisons of different GFs for healing rate, only EGF showed a statistically significant difference when compared to FGF (RR = 2.0, 95% CI = 1.16–3.59). The league table comparing the healing rates of various treatments is shown in **Figure 3**.

### Healing time

Compared to SOC, EGF (MD = −24.94, 95% CI = −40.76 to −9.38) and PRP (MD = −16.92, 95% CI = −26.15 to −7.09) significantly reduced healing time, with statistically significant differences. No statistically significant differences were found in the comparisons of healing time among different GFs. The league table comparing healing times across various treatments is shown in **Supplementary Figure S2**.

### Reduction in ulcer area

Compared to SOC, PDGF (MD = −9.91, 95% CI = −17.79 to −2.04) significantly reduced ulcer area, with a statistically significant difference. No statistically significant differences were observed in the comparisons of ulcer area reduction among different GFs. The league table comparing the reduction in ulcer area across various treatments is shown in **Supplementary Figure S3**.

### AEs

Compared to SOC, PRP (RR = 0.27, 95% CI = 0.09–0.79) significantly reduced the incidence of AEs. VEGF was associated with a significantly increased incidence of AEs compared to EGF, FGF, PDGF, PRP, and SOC, with all differences being statistically significant. No statistically significant differences were observed in other treatment comparisons. The league table comparing the incidence of AEs across various treatments is shown in **Supplementary Figure S4**.

### Amputation rate

Compared to SOC, PRP (RR = 0.17, 95% CI = 0.01–0.61) significantly reduced the amputation rate, with a statistically significant difference. No statistically significant differences were observed in the comparisons of amputation rates among different GFs. The league table comparing amputation rates across various treatments is shown in **Supplementary Figure S5**.

### SUCRA

Network meta-analysis enables the ranking of interventions through the calculation of SUCRA, which ranges from 0 to 100%. A higher SUCRA value corresponds to a better ranking position, indicating that the intervention not only demonstrates superior efficacy but also has better safety.

**Figure 4:**
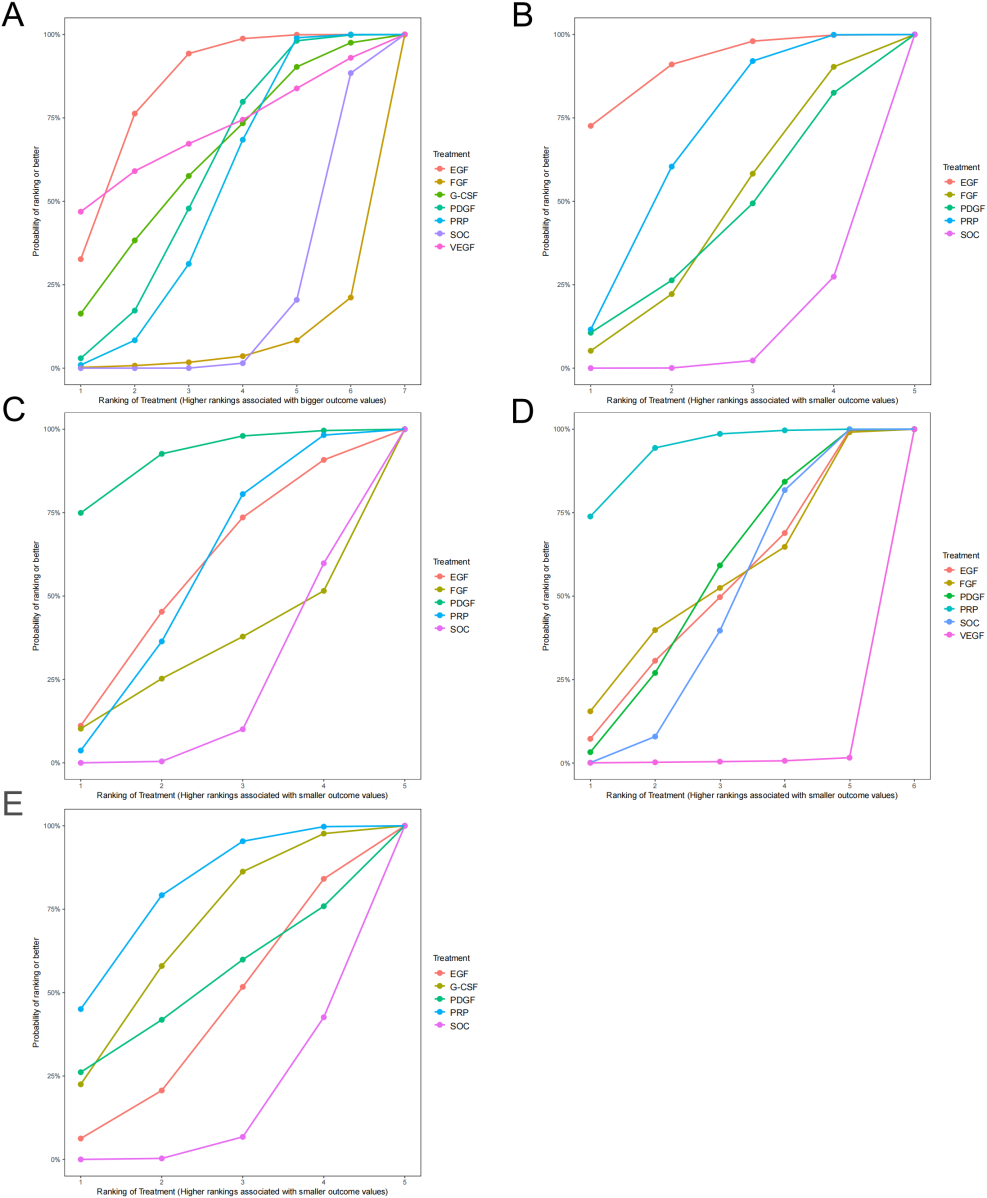
Cumulative ranking curves (SUCRA plots) for **(A)** healing rate, **(B)** healing time, **(C)** ulcer area reduction, **(D)** incidence of adverse events, and **(E)** amputation rate. The surface under the cumulative ranking curve (SUCRA) indicates the relative ranking probability of each treatment, with higher SUCRA values representing better performance for positive outcomes and lower risk for negative outcomes. PRP, platelet-rich plasma; SOC, standard of care; FGF, fibroblast growth factor; PDGF, platelet-derived growth factor; G-CSF, granulocyte colony-stimulating factor; EGF, epidermal growth factor; VEGF, vascular endothelial growth factor.

Detailed SUCRA values for healing rate, healing time, ulcer area reduction, incidence of AEs, and amputation rate are shown in **Table 2**. **Figures 4A–E** present the cumulative ranking probability plots for healing rate, healing time, ulcer area reduction, incidence of AEs, and amputation rate, respectively. EGF ranks first in healing rate and healing time, second to PDGF in ulcer area reduction, and PRP ranks first in amputation rate and incidence of AEs. Therefore, in terms of healing rate, all GFs except FGF ranked higher than SOC, with EGF, PDGF, and PRP showing significantly better outcomes than SOC. Regarding healing time, all GFs ranked higher than SOC, with EGF and PRP demonstrating statistically significant improvements. For ulcer area reduction, all GFs outperformed SOC, with PDGF showing a significant advantage. Among the GFs, EGF appears to be the most effective. Compared to SOC, only VEGF was associated with a significant increase in AEs, while other GFs did not show a significant difference. PRP was associated with the lowest incidence of AEs and the lowest amputation rate.

**Table 2:** SUCRA values and ranks of efficacy outcomes for different interventions in diabetic foot ulcer treatment.

Interventions	Healing rate		Healing time		Ulcer reduction area		Adverse events		Amputation rate	
	SUCRA (%)	Rank	SUCRA (%)	Rank	SUCRA (%)	Rank	SUCRA (%)	Rank	SUCRA (%)	Rank
EGF	84	1	90	1	55	2	51	4	41	4
PDGF	58	4	42	4	91	1	55	2	51	3
PRP	51	5	66	2	55	3	93	1	80	1
G-CSF	62	3	–	–	–	–	–	–	66	2
FGF	6	7	44	3	31	4	54	3	–	–
VEGF	71	2	–	–	–	–	1	6	–	–
SOC	18	6	7	5	18	5	46	5	12	5

PRP, platelet-rich plasma; SOC, standard of care; FGF, fibroblast growth factor; PDGF, platelet-derived growth factor; G-CSF, granulocyte colony-stimulating factor; EGF, epidermal growth factor; VEGF, vascular endothelial growth factor; AEs, adverse events; SUCRA; surface under the cumulative ranking curve.

### Sensitivity analysis

After excluding high-risk studies, the healing rate was reassessed. The results showed no significant difference from the findings before excluding the high-risk studies, and the rankings remained unchanged. This indicates that the results of the primary outcome measures in this study are reliable.

## Discussion

The wound healing in patients with DFU is influenced by factors such as vascular abnormalities, neuropathy, and inflammation stasis, which obstruct the healing process (69). Additionally, the levels of GFs in the wound are low, further exacerbating the difficulty of healing (70). GFs play specific roles in regulating the healing process, and their positive effects on diabetic wound treatment have been well established (71). As a novel therapeutic approach, GFs are considered an effective means for treating DFU, although consensus has not yet been reached (6). Current studies mainly focus on comparing the efficacy of a single GF with SOC, leading to a lack of evidence regarding the head-to-head comparison of different GFs in terms of their effectiveness and safety in treating DFU. Therefore, the aim of our study is to evaluate the efficacy and safety of different GFs in treating DFU, providing more evidence-based medicine for the treatment of DFU.

To the best of our knowledge, this study is the first to comprehensively assess the efficacy and safety of different GFs in the treatment of DFU. Two previous network meta-analyses (11, 12) only reported healing rate as an outcome. To determine both efficacy and safety, we further evaluated healing time, ulcer area reduction, AEs, and amputation rate. The results of our primary outcome measures are consistent with those of the above-mentioned network meta-analyses, both showing that, compared to SOC, EGF, PDGF, and PRP significantly improved the healing rate of DFU. Among these, EGF may be the most effective GF for healing rate. Our study suggested that almost all GFs demonstrated superior performance to SOC in terms of healing rate, healing time, and ulcer area reduction, with EGF emerging as the most potentially effective GF. Except for VEGF, which significantly increased AEs, other GFs did not show a significant increase in AEs. PRP was associated with the lowest incidence of AEs and the lowest amputation rate. After excluding high-risk studies, we re-evaluated the healing rate and found no significant changes in the results, with the ranking of interventions remaining consistent, indicating that the primary outcome was robust and reliable. However, because of the limited number of studies reporting secondary outcomes, sensitivity analyses could not be performed, which restricts further validation of these results.

In terms of healing rate and healing time, EGF shows the greatest consistency across multiple RCTs (24, 29, 32, 38, 39, 44, 50, 51, 54, 56, 57), with its efficacy significantly outperforming SOC. EGF promotes the proliferation and migration of keratinocytes, enhances collagen synthesis, and accelerates the epithelialization process (72, 73). Compared to FGF, EGF has a significant advantage in healing rate, which may be related to the lower levels of EGF in diabetic foot tissue (74). The supplementation of exogenous EGF directly promotes wound healing and accelerates tissue repair by inhibiting non-enzymatic glycosylation through a feedback mechanism (49). On the other hand, FGF, as a competitive antagonist of advanced glycation end products, typically requires higher concentrations to improve wound healing and its effects appear more slowly, leading to a longer treatment cycle (75–77). Additionally, it is noteworthy that in our network meta-analysis, FGF’s healing rate ranking was lower than SOC. In the relevant RCT, FGF did not significantly improve the healing rate of DFU compared to SOC (19). Since the number of RCTs involving FGF is limited, this result still needs to be further verified through more multi-center, high-quality, and long-term follow-up RCTs.

PDGF significantly outperforms SOC in ulcer area reduction. During the wound healing process, PDGF plays a key role by promoting the proliferation and migration of inflammatory cells, aiding in debridement, and stimulating the formation of granulation tissue (78–80). Additionally, PDGF promotes angiogenesis and the differentiation of myofibroblasts, which accelerates the healing of diabetic wounds (81). Clinical trials have shown that PDGF significantly increases the healing speed of diabetic wounds and greatly enhances the probability of complete healing (7, 82–84).

PRP significantly outperforms SOC in terms of incidence of AEs and amputation rate. As an autologous treatment, PRP effectively avoids immune rejection and allergic reactions, reduces the risk of infection, and, owing to its excellent biocompatibility, typically does not cause severe side effects (85). In reducing the amputation rate, PRP accelerates wound healing, improves local blood supply, regulates inflammatory responses, and controls infections, successfully preventing the deterioration of DFU, reducing the occurrence of complications, and significantly lowering the amputation risk, thereby improving treatment outcomes and prognosis (86).

Despite the variety of GFs, the products currently entering clinical trials remain relatively limited (87). This study provides more evidence-based medical evidence for the treatment of DFU and further validates the application prospects of GFs in this field. Future research should focus on the impact of different doses of GFs on treatment outcomes and compare the efficacy and cost-effectiveness of different GFs. Additionally, the combined use of different GFs or GFs with other treatment modalities (such as stem cell transplantation and anti-inflammatory drugs) may offer more promising treatment options for DFU (69, 88). Therefore, conducting more clinical trials to evaluate the efficacy and feasibility of these combination therapies is crucial.

Our study also has certain limitations: (1) Network meta-analysis can be affected by confounding factors and cannot fully replace clinical trials that directly compare treatments. Therefore, the conclusions of this study still require further confirmation through direct comparisons of different GFs. (2) The included studies had varying patient ages, wound duration, and drug dosages, and because of the limited number of studies included, we were unable to perform in-depth subgroup analyses. Owing to the varying quality of the studies included, we were unable to conduct subgroup analyses based on the type of ulcer (neuropathic, vascular, or mixed) or the severity of the ulcers in patients with DFU. The reporting time for outcome measures in the included studies was inconsistent, and thus, we were unable to conduct analyses based on specific time points. These factors may compromise the accuracy of the results. For instance, patients in different age groups may respond differently to GF therapy, and the type and severity of ulcers can significantly influence treatment outcomes. Furthermore, variations in GF dosage may lead to differences in therapeutic efficacy. Consequently, the absence of subgroup analyses may obscure treatment effect differences across specific patient subgroups, thereby limiting a comprehensive evaluation of the intervention’s overall effectiveness. (3) Although SOC in the included studies was based on guideline recommendations, the specific types varied, which may have influenced the efficacy evaluation of different GFs.

In summary, almost all GFs outperformed SOC in terms of healing rate, healing time, and ulcer area reduction, with EGF appearing to be the most efficacious GF. Except for VEGF, which significantly increased the incidence of AEs, other GFs did not show significant effects on AEs, suggesting a favorable safety profile. Among them, PRP was associated with the lowest incidence of AEs and the lowest amputation rate. After excluding high-risk studies, the changes in healing rate were not significant, and the ranking of interventions remained consistent, supporting the robustness of the results. However, the limited availability of data for secondary outcomes restricted the ability to fully assess their reliability. Because of the limitations of the current study, the conclusions still require validation through a large number of high-quality RCTs that directly compare different GFs with SOC or compare different GFs in the treatment of DFU.

## Data Availability

The original contributions presented in the study are included in the article/**Supplementary Material**. Further inquiries can be directed to the corresponding author.
