# Supplementary material for: Comparison of the efficacy and safety of different growth factors in the treatment of diabetic foot ulcers: an updated network meta-analysis
Source: Front Endocrinol (Lausanne). 2025 Jun 25;16:1614597. doi: 10.3389/fendo.2025.1614597 (PMC12237656; doi:10.3389/fendo.2025.1614597)
Supplement: Supplementary file 1 [file DataSheet1.docx]

**Supplementary File S1.** Detailed search strategies for each database

**PubMed**

#1 "Diabetic Foot"[Mesh]

#2 "Diabetic Foot" OR "Diabetic Feet" OR "Diabetic Foot Ulcer")*

#3 #1 OR #2

#4 diabetic OR diabetes

#5 foot OR feet

#6 #4 AND #5

#7 wound*

#8 #4 AND #7

#9 #3 OR #6 OR #8

#10 "Growth factor*"

#11 "Epidermal Growth Factor"[Mesh]

#12 "Epidermal Growth Factor*" OR EGF

#13 #11 OR #12

#14 "Fibroblast Growth Factors"[Mesh]

#15 "Fibroblast Growth Factor*" OR FGF

#16 #14 OR #15

#17 "Platelet-Derived Growth Factor"[Mesh]

#18 "Platelet-Derived Growth Factor" OR "Platelet Derived Growth Factor*" OR PDGF

#19 #17 OR #18

#20 "Vascular Endothelial Growth Factors"[Mesh]

#21 "Vascular Endothelial Growth Factor*" OR VEGF

#22 #20 OR #21

#23 "Transforming Growth Factors"[Mesh]

#24 "Transforming Growth Factor*" OR TGF

#25 #23 OR #24

#26 "Platelet-Rich Plasma"[Mesh]

#27 "Platelet-Rich Plasma" OR "Platelet Rich Plasma" OR PRP

#28 #26 OR #27

#29 "Granulocyte Colony-Stimulating Factor*"

#30 "Granulocyte Colony Stimulating Factor*" OR G-CSF

#31 #29 OR #30

#32 #10 OR #13 OR #16 OR #19 OR #22 OR #25 OR #28 OR #31

#33 #9 AND #32

**Embase**

#1 'diabetic foot'/exp OR 'diabetic foot' OR 'diabetic feet' OR 'diabetic foot ulcer*'

#2 'diabetic' OR 'diabetes'

#3 'foot' OR 'feet'

#4 #2 AND #3

#5 wound*

#6 #2AND #5

#7 #1 OR #4 OR #6

#8 'growth factor*'

#9 'epidermal growth factor'/exp OR 'epidermal growth factor' OR egf

#10 'fibroblast growth factors'/exp OR 'fibroblast growth factor*' OR fgf

#11 'platelet-derived growth factor'/exp OR 'platelet-derived growth factor' OR 'platelet derived growth factor*' OR pdgf

#12 'vascular endothelial growth factors'/exp OR 'vascular endothelial growth factor*' OR vegf

#13 'transforming growth factors'/exp OR 'transforming growth factor*' OR tgf

#14 'platelet-rich plasma'/exp OR 'platelet-rich plasma' OR 'platelet rich plasma' OR prp

#15 'granulocyte colony-stimulating factor*' OR 'granulocyte colony stimulating factor*' OR 'g csf'

#16 #8 OR #9 OR #10 OR #11 OR #12 OR #13 OR #14 OR #15

#17 #7 AND #16

**The Cochrane Library**

#1 MeSH descriptor: [Diabetic Foot] explode all trees

#2 ("Diabetic Foot") OR ("Diabetic Feet") OR ("Diabetic Foot Ulcer*")

#3 #1 OR #2

#4 'diabetic' OR 'diabetes'

#5 'foot' OR 'feet'

#6 #4 AND #5

#7 wound*

#8 #4 AND #7

#9 #3 OR #6 OR #8

#10 ("Growth factor*")

#11 ("Epidermal Growth Factor") OR ("Epidermal Growth Factor*") OR (EGF)

#12 ("Fibroblast Growth Factors") OR ("Fibroblast Growth Factor*") OR (FGF)

#13 ("Platelet-Derived Growth Factor") OR ("Platelet Derived Growth Factor*") OR (PDGF)

#14 ("Vascular Endothelial Growth Factors") OR ("Vascular Endothelial Growth Factor*") OR (VEGF)

#15 ("Transforming Growth Factors") NOT ("Transforming Growth Factor*") OR (TGF)

#16 ("Platelet-Rich Plasma") OR ("Platelet Rich Plasma") OR (PRP)

#17 ("Granulocyte Colony-Stimulating Factor*") OR ("Granulocyte Colony Stimulating Factor*") OR (G-CSF)

#18 #10 OR #11 OR #12 OR #13 OR #14 OR #15 OR #16 OR #17

#19 #9 AND #18

**Web of Science**

#1 "Diabetic Foot"(Topic) OR "Diabetic Feet"(Topic) OR "Diabetic Foot Ulcer*"(Topic)

#2 diabetic (Topic) OR diabetes (Topic)

#3 foot (Topic) OR feet (Topic)

#4 #2 AND #3

#5 wound*(Topic)

#6 #2 AND #5

#7 #1 OR #4 OR #6

#8 "Growth factor*"(Topic)

#9 "Epidermal Growth Factor"(Topic) OR "Epidermal Growth Factor*"(Topic) OR EGF (Topic)

#10 "Fibroblast Growth Factors"(Topic) OR "Fibroblast Growth Factor*"(Topic) OR FGF(Topic)

#11 "Platelet-Derived Growth Factor"(Topic) OR "Platelet Derived Growth Factor*"(Topic) OR PDGF(Topic)

#12 "Vascular Endothelial Growth Factors"(Topic) OR "Vascular Endothelial Growth Factor*"(Topic) OR VEGF(Topic)

#13 "Transforming Growth Factors"(Topic) OR "Transforming Growth Factor*"(Topic) OR TGF(Topic)

#14 "Platelet-Rich Plasma"(Topic) OR "Platelet Rich Plasma"(Topic) OR PRP (Topic)

#15 "Granulocyte Colony-Stimulating Factor*"(Topic) OR "Granulocyte Colony Stimulating Factor*"(Topic) OR G-CSF(Topic)

#16 #8 OR #9 OR #10 OR #11 OR #12 OR #13 OR #14 OR #15

#17 #7 AND #16

**Supplementary Figure S1.** Risk of bias assessment results


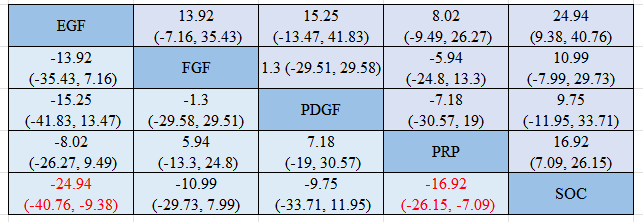


**Supplementary Figure S2.** League table comparing healing time among different treatment interventions. Values represent mean differences (MDs) with 95% confidence intervals (CIs) for each pairwise comparison, where the treatment in the column is compared to the treatment in the row. If the MD is less than 0 and the difference is statistically significant, the treatment in the column is superior to the treatment in the row. Statistically significant results (p < 0.05) are highlighted in bold red font. PRP, platelet-rich plasma; SOC, standard of care; FGF, fibroblast growth factor; PDGF, platelet-derived growth factor; EGF, epidermal growth factor.


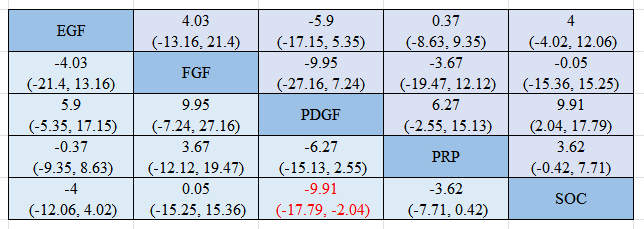


**Supplementary Figure S3.** League table comparing ulcer area reduction among different treatment interventions. Values represent mean differences (MDs) with 95% confidence intervals (CIs) for each pairwise comparison, where the treatment in the column is compared to the treatment in the row. If the MD is less than 0 and the difference is statistically significant, the treatment in the column is superior to the treatment in the row. Statistically significant results (p < 0.05) are highlighted in bold red font. PRP, platelet-rich plasma; SOC, standard of care; FGF, fibroblast growth factor; PDGF, platelet-derived growth factor; EGF, epidermal growth factor.


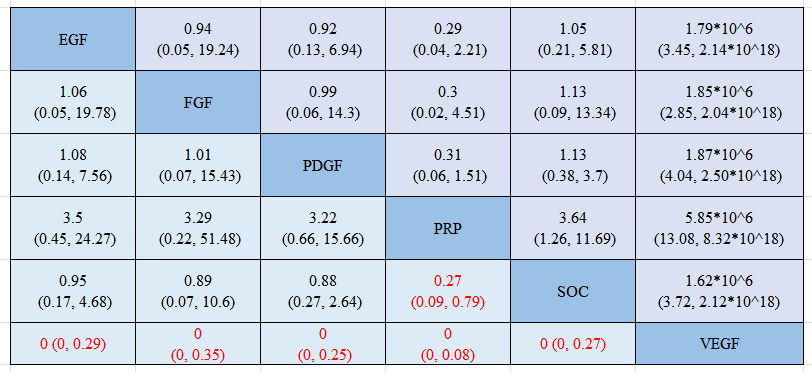


**Supplementary Figure S4.** League table comparing the incidence of adverse events (AEs) among different treatment interventions. Values represent relative risks (RRs) with 95% confidence intervals (CIs) for each pairwise comparison, where the treatment in the column is compared to the treatment in the row. If the RR is less than 1 and the difference is statistically significant, the treatment in the column is superior to the treatment in the row. Statistically significant results (p < 0.05) are highlighted in bold red font. PRP, platelet-rich plasma; SOC, standard of care; FGF, fibroblast growth factor; PDGF, platelet-derived growth factor; EGF, epidermal growth factor; VEGF, vascular endothelial growth factor.


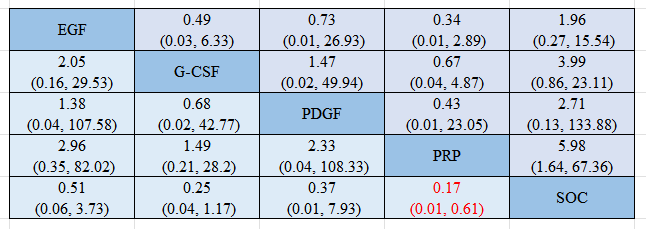
**Supplementary Figure S5.** League table comparing amputation rates among different treatment interventions. Values represent relative risks (RRs) with 95% confidence intervals (CIs) for each pairwise comparison, where the treatment in the column is compared to the treatment in the row. If the RR is less than 1 and the difference is statistically significant, the treatment in the column is superior to the treatment in the row. Statistically significant results (p < 0.05) are highlighted in bold red font. PRP, platelet-rich plasma; SOC, standard of care; PDGF, platelet-derived growth factor; G-CSF, granulocyte colony-stimulating factor; EGF, epidermal growth factor.
